# The dual diversity crisis in alzheimer's disease research: why neuroimaging biomarkers and clinical trials keep failing

**DOI:** 10.1007/s11357-026-02400-x

**Published:** 2026-07-06

**Authors:** Thorsten Rudroff

**Affiliations:** Turku PET Centre, University of Turku and Turku University Hospital, Turku, Finland

**Keywords:** Alzheimer’s disease, Neuroimaging biomarkers, WYHU research

## Abstract

Alzheimer's disease clinical trials have failed at a rate exceeding 99% over two decades, and neuroimaging biomarkers developed in discovery cohorts have repeatedly disappointed in validation and clinical application. Standard explanations address symptoms rather than structural causes. I argue that Alzheimer's disease research is caught in a Dual Diversity Crisis: the simultaneous neglect of population diversity and individual neurobiological heterogeneity as compounding validity threats. The first dimension concerns the systematic over-reliance on Western, young, healthy, and university-affiliated (WYHU) research samples, from which biomarker normative standards are derived and then applied universally to a demographically distinct clinical population. The second concerns the erasure, through group-level analysis, of the substantial neurobiological heterogeneity that exists within any sample regardless of its demographic composition. The critical structural insight is that these two problems do not add; they multiply. A WYHU-derived group average is doubly unrepresentative: it misrepresents the target population demographically and conceals the individual variance that exists even within that already-unrepresentative sample. FDG-PET evidence demonstrates that biological sex accounts for approximately 30 times more metabolic variance than diagnostic category in patients with equivalent symptom profiles, directly challenging the construct validity of threshold-based trial inclusion. Correcting the Dual Diversity Crisis requires treating demographic diversity and individual neurobiological characterization as primary design parameters, not post-hoc corrections. Until this reconceptualization occurs, translational failure in Alzheimer's disease research remains the structurally expected outcome.

## Introduction

Alzheimer's disease research has absorbed more funding and scientific talent than almost any other neurological condition of our era, yet its translation record is marked by serial failure. More than 99% of clinical trials over the past two decades have failed [1]. Neuroimaging biomarkers that appear robust in discovery cohorts have repeatedly disappointed in validation studies and clinical application. The amyloid cascade hypothesis, despite generating a generation of therapeutics, produced decades of largely negative trial outcomes before modest efficacy signals emerged with lecanemab and donanemab, and even those signals arrived amid unresolved questions about who benefits, by how much, and at what risk.

Standard explanations for this record are well known: underpowered studies, publication bias, surrogate endpoints, inadequate patient stratification, and the fundamental difficulty of intervening in a disease that may begin decades before clinical symptoms. These explanations are not wrong, but they address symptoms rather than a structural cause. I argue that Alzheimer's disease neuroimaging and clinical trial research is caught in what I term the Dual Diversity Crisis: the simultaneous neglect of population diversity and individual neurobiological heterogeneity as compounding validity threats. Neither problem alone accounts for the scale of translational failure. Together, they create conditions under which biomarker invalidity and trial failure are not statistical accidents but structurally overdetermined outcomes.

This argument does not displace the other recognized contributors to translational failure, including suboptimal intervention timing relative to disease onset, incomplete target validation, the challenge of blood–brain barrier penetration for candidate therapeutics, endpoint insensitivity to early-stage biological change, network-level neurodegeneration that extends beyond primary amyloid and tau pathology, vascular contributions to cognitive impairment, and the high prevalence of mixed pathologies in older adults. These factors are real and consequential. The claim advanced here is that the Dual Diversity Crisis is structurally prior to them: a research architecture that begins with a demographically unrepresentative sample and erases individual neurobiological variance through group-level analysis will propagate those errors through every downstream stage, compounding the impact of every other source of translational failure. Correcting the Dual Diversity Crisis does not guarantee success; failing to correct it structurally forecloses it.

## The dual diversity crisis: population and individual dimensions

The empirical foundation of Alzheimer's neuroimaging rests predominantly on samples that are Western, young for their diagnostic category, healthy in terms of comorbidity burden, and university-affiliated in terms of recruitment context, a constellation I abbreviate as WYHU. The Alzheimer's Disease Neuroimaging Initiative (ADNI), the primary data infrastructure for biomarker development internationally, enrolled participants who were predominantly white and college-educated across its foundational phases, with minimal representation from Black, Hispanic, Asian, and indigenous populations [2]. This is not primarily an equity problem, though it is certainly that. It is a validity problem. Neurobiological parameters treated as universal reference values, including amyloid burden thresholds, tau staging cutoffs, and hippocampal volume norms, were derived from samples that do not represent the population to which they are applied. Ancestral background modifies amyloid deposition rates, APOE genotype frequencies, vascular comorbidity burden, and inflammatory profiles in ways that WYHU-derived norms cannot accommodate [3]. The A/T/N framework, which classifies individuals by amyloid, tau, and neurodegeneration biomarker status and now serves as the organizational backbone of Alzheimer's research and trial design, represents a genuine conceptual advance [4]. But its normative parameters carry the full weight of the WYHU problem. Applying the framework universally does not solve biomarker invalidity; it institutionalizes and scales it.

The WYHU abbreviation extends the WEIRD critique of Henrich and colleagues [5], who documented the systematic over-reliance on Western, Educated, Industrialized, Rich, and Democratic samples in behavioral science, to the neuroimaging and clinical trial context where the validity consequences are more directly tied to biomedical harm. Before proceeding, a brief conceptual disambiguation is necessary. Demographic diversity refers to the representativeness of research populations across ancestry, sex, age, socioeconomic position, and geographic origin. Individual neurobiological heterogeneity refers to variance in biological parameters within any population, regardless of its demographic composition. Precision medicine refers to the clinical aspiration to match intervention to individual biological profile. Subgroup stratification refers to the analytic practice of disaggregating outcomes by defined group characteristics. Individualized neurobiology refers to the empirical recognition that no two brains, even within a demographic stratum, express disease in identical ways. These concepts overlap but are not interchangeable. The Dual Diversity Crisis addresses the first two specifically: it identifies demographic narrowness and heterogeneity erasure as compounding validity threats at the level of research design, prior to any stratification or precision medicine implementation. Correcting it is a precondition for the other aspirations to be achievable.

The second component of the Dual Diversity Crisis concerns not who is studied but what is found within any sample, regardless of its demographic composition. Alzheimer's disease is not a single neurobiological entity. Neuropathological and neuroimaging evidence has established substantial subtypes within the clinical diagnosis: hippocampal-sparing, limbic-predominant, and posterior cortical atrophy variants differ in tau spreading trajectories, progression rates, and likely therapeutic responses [6, 7]. Amyloid burden shows a well-documented dissociation from cognitive symptoms: substantial accumulation is present in a significant proportion of cognitively unimpaired older adults, reflecting genuine individual heterogeneity in the biological relationship between amyloid deposition and neurotoxic cascade initiation [8]. Biological sex compounds this heterogeneity further. Women account for approximately two-thirds of Alzheimer's disease cases. Female sex is associated with greater tau burden at equivalent amyloid levels, distinct hippocampal atrophy trajectories, and different APOE4 penetrance profiles [9, 10]. These are not minor quantitative differences; they represent biologically distinct disease trajectories grouped under a single diagnostic label and evaluated against normative standards derived predominantly from mixed-sex or male-weighted discovery cohorts. When group-level analysis averages across this degree of internal heterogeneity, the reported treatment effect or biomarker association is a central tendency statistic drawn from a biologically heterogeneous distribution.

Ancestry introduces a further layer of heterogeneity that is incompletely captured even in more demographically diverse cohorts. APOE4 confers differential risk depending on ancestral background: its effect size in African American populations is attenuated relative to non-Hispanic white populations, while APOE2-related protection shows similar population specificity [11]. Amyloid accumulation trajectories and tau staging profiles show measurable variation across ancestry groups that WYHU-derived normative thresholds will systematically misclassify. Vascular contributions to cognitive impairment add a further axis of heterogeneity: subcortical white matter pathology, lacunar infarcts, and cerebrovascular reactivity alterations co-occur with Alzheimer's pathology at rates that vary substantially by ancestry, sex, and comorbidity burden, and that alter the neurobiological context in which amyloid and tau thresholds are interpreted [12]. Mixed dementia pathology, present in a substantial proportion of older adults at autopsy, means that a diagnosis of Alzheimer's disease frequently indexes a neurobiologically heterogeneous combination of proteinopathies whose relative contributions vary across individuals [13]. At the global epidemiological level, Alzheimer's disease incidence, prevalence, vascular risk factor burden, and demographic aging structure differ substantially across world regions, and the applicability of biomarker thresholds derived from North American and European cohorts to populations in sub-Saharan Africa, South and East Asia, or Latin America cannot be assumed [14]. The ADNI model has been enormously productive, but its success underscores the need for analogous initiatives in low- and middle-income countries with population-representative recruitment.

## How the two dimensions compound

The critical insight of the Dual Diversity framework is that these two problems do not add; they multiply. A study conducted on a WYHU sample will produce group-level findings that are doubly unrepresentative: first, because the sample does not reflect the demographic reality of the Alzheimer's population; and second, because the group average conceals the neurobiological variance that exists even within that already-unrepresentative sample.

Consider a trial that recruits amyloid-positive individuals meeting an ADNI-derived threshold. The recruited sample will be skewed toward the demographic profile of ADNI. Within that already-narrowed sample, amyloid positivity will group together individuals across the full range of neurobiological subtypes, tau staging profiles, sex-specific trajectory patterns, and comorbidity burdens. The trial then tests an intervention against an average outcome in a population that was never representative, and whose internal heterogeneity ensures that the average outcome is a statistical artifact of aggregation. Under these conditions, trial failure is not an unfortunate outcome. It is the structurally expected result.

This compounding logic also explains why biomarkers that perform well in discovery cohorts fail in validation. Discovery cohorts, drawn from academic medical centers with high research engagement, are particularly homogeneous in both demographic and neurobiological terms. Within that constrained variance, a biomarker association can appear strong. When the same biomarker is applied to a validation cohort with greater demographic diversity and a less controlled recruitment context, both sources of variance expand simultaneously. The association attenuates, the threshold misclassifies, and the finding fails to replicate. The problem is routinely attributed to the validation cohort's noisiness rather than to the discovery cohort's artificial homogeneity.

## Cross-diagnostic evidence: symptom equivalence does not imply neurobiological equivalence

A directly relevant empirical demonstration comes from FDG-PET neuroimaging comparing patients with multiple sclerosis and patients with Long COVID who presented with clinically equivalent fatigue severity on validated symptom scales [15]. Despite identical conscious reports, the two groups showed qualitatively distinct frontal-striatal glucose metabolic patterns. More strikingly, biological sex accounted for approximately 30 times more variance in globus pallidus metabolic activity than diagnostic category itself.

The implications for Alzheimer's disease are direct. If two patients sharing the same symptom score and the same clinical profile are neurobiologically distinct in ways entirely obscured by symptom-level equivalence, there is no principled basis for supposing that amyloid positivity above a given cutoff represents a neurobiologically homogeneous treatment target. Threshold-based trial inclusion defines a statistical region of a distribution; it does not define a neurobiologically equivalent category. Treating it as though it does constitute a construct validity failure at the point of trial design, one that cascades irreversibly through every subsequent analysis.

## Implications for research architecture

Acknowledging the Dual Diversity Crisis does not require abandoning the neuroimaging biomarker enterprise. It requires redesigning its foundational architecture.

At the level of cohort construction, demographic diversity must be treated as a scientific validity requirement, not an ethical addendum. This requires prospective recruitment strategies with genuine ancestral, socioeconomic, and geographic breadth, combined with disaggregated reporting by demographic subgroup with sufficient within-group sample sizes to support meaningful inference. Cosmetic oversampling that leaves within-group heterogeneity statistically unresolvable does not address the problem.

At the level of biomarker development, ancestry-specific and sex-stratified reference atlases for amyloid PET, tau PET, and structural MRI are a precondition for validity, not a future aspiration. The current practice of applying WYHU-derived normative standards universally generates classification errors that are not random but systematically biased in predictable demographic directions.

At the level of trial design, biomarker-based stratification must move beyond binary positivity thresholds to incorporate the dimensional neurobiological profile of individual participants, including tau staging, subtype classification, sex-specific trajectory indicators, and vascular comorbidity burden. The A/T/N framework requires extension: its three biological dimensions contain no information about the demographic and individual-level sources of variance that determine how those processes manifest and respond to intervention. A diversity dimension, in both senses articulated here, is biologically necessary for the framework's clinical validity.

Several concrete directions follow from this reconceptualization. First, federated multinational neuroimaging datasets, in which data from demographically diverse cohorts across multiple world regions are harmonized and made jointly analyzable while preserving within-site biological specificity, offer a practical path toward the demographic breadth that single-site cohorts cannot achieve. Federated approaches avoid the pooling artifacts that can arise when raw demographic heterogeneity is treated as statistical noise rather than as biological signal. Second, AI-based neurobiological subtype clustering applied to multimodal imaging data, including amyloid PET, tau PET, structural MRI, and FDG-PET, can reveal data-driven subtypes within currently heterogeneous diagnostic categories, making the individual neurobiological heterogeneity visible rather than averaged away. These clusters can then become the units of stratification for trial enrollment and biomarker threshold derivation, replacing the current practice of grouping biologically dissimilar individuals under shared diagnostic labels. Third, adaptive trial enrichment strategies, in which enrollment criteria are updated mid-trial based on emerging biomarker-response relationships within pre-specified subgroups, can partially compensate for initial heterogeneity if the enrichment criteria are themselves grounded in neurobiologically meaningful dimensions rather than demographic proxies. Fourth, longitudinal resilience and cognitive reserve metrics, which capture the individual trajectory of biological change over time rather than a single cross-sectional threshold status, address the amyloid-cognition dissociation problem directly: they permit the identification of individuals whose biological trajectories signal imminent cognitive decline, rather than those who simply cross a normative cutoff derived from a demographically unrepresentative reference population. Fifth, multimorbidity-informed biomarker frameworks that explicitly model vascular pathology, metabolic burden, inflammatory profiles, and mixed proteinopathies as co-determinants of neuroimaging signal, rather than as covariates to be statistically removed, would move the field toward the biological realism that clinical translation requires. These directions are technically feasible with existing methods. What they require is the conceptual reorientation that the Dual Diversity framework provides: the recognition that demographic diversity and individual neurobiological characterization are not peripheral quality improvements but constitutive requirements of valid science.

## Conclusion

The long record of failure in Alzheimer's disease neuroimaging and clinical trials is not a story of insufficient scientific effort or inadequate investment. It is a story of a field that built its knowledge infrastructure on a systematically unrepresentative empirical base and compounded that problem by analyzing the resulting data in ways that erase the very heterogeneity most relevant to clinical translation.

The Dual Diversity Crisis is structural to this failure, not peripheral to it. The tools for correction exist. What has been lacking is the framework for recognizing why they are scientifically necessary rather than merely ethically desirable. That framework is now available. The cost of continuing without it is already visible in two decades of translational failure.
